# Why?

**Published:** 1880-02

**Authors:** 


WHY will a sensible man attempt to enter an omnibus on a rainy day with the large end of his umbrella foremost?

Why do ladies from the country and un-self-possessed ladies from the town, walk the streets with one end of their parasol stuck in the mouth or near the chin? We knew a lady to be run against by a man in a hurry, the end of the parasol was driven against a tooth, knocked it out, and passing on, pierced the roof of the mouth.

Why are points made to parasols and umbrellas? A year or two ago a person was walking along the streets of New York rapidly, another being ahead of him with the sharp point of his umbrella sticking out under his arm or over his shoulder, and stopping suddenly, the point entered the eye of the man following, causing a wound which terminated in death.

Why do the architects of New York persist in having great wooden cornices to their buildings? They are children's make-believe; resemble nothing under the sun but themselves; are not only useless, but are eminently dangerous, in case of fire. I have seen a quarter million of dollars destroyed in two hours of a winter's night by the cornices taking fire from a building on the opposite side of the street.

Why will a gentleman who pretends to any degree of personal neatness and deportment, especially if he has cough, cold or consumption, evacuate his mouth and nose on the side-walk in preference to stepping to the gutter?

Why does a gentleman or lady, after service, stop another in the church aisle and engage in conversation, thus arresting all in their rear?

Why do nine women out of ten have the *vis-inertia* of the Cordilleras when another person wishes to occupy a vacant seat beside them in an omnibus, pew, or rail-car?

Why do sensible people often sit down to table and begin to eat "*when they don't feel like it,*" and *force it down*, when they know that a pig could not be hired to such a performance, to say nothing of the loss of that much food to the world, which many a poor brother in humanity would be glad to get?

